# QKI 6 ameliorates CIRI through promoting synthesis of triglyceride in neuron and inhibiting neuronal apoptosis associated with SIRT1‐PPARγ‐PGC‐1α axis

**DOI:** 10.1002/brb3.2271

**Published:** 2021-07-05

**Authors:** Rui Liu, Hongzeng Li, Jingyuan Deng, Qunqiang Wu, Chunhua Liao, Qun Xiao, Qi Chang

**Affiliations:** ^1^ Department of Rehabilitation Tangdu Hospital Fourth Military Medical University Xi'an PR China; ^2^ Department of Gerontology Tangdu Hospital Fourth Military Medical University Xi'an PR China; ^3^ Department of Encephalology the First Affiliated Hospital Medical School of Xi'an Jiaotong University Xi'an PR China; ^4^ Department of Orthopaedics Xijing Hospital Fourth Military Medical University Xi'an PR China; ^5^ Department of Orthopaedics The 150th Central Hospital of Chinese People's Liberation Army Luoyang PR China

**Keywords:** cerebral ischemia/reperfusion injury, middle cerebral artery occlusion, neuronal apoptosis, Quaking 6, sirtuin 1, synthesis of triglyceride

## Abstract

**Background:**

The stroke induced by ischemia of brain remains high incidence and death rate. The study wanted to confirm the effects of Quaking 6 (QKI 6) on the protection role in neurons of rat model of cerebral ischemia/reperfusion injury (CIRI).

**Material and methods:**

The rat model with CIRI induced by middle cerebral artery occlusion was well established and rat neurons were isolated to characterize the effects of QKI 6 mediated by sirtuin 1 (SIRT1) on synthesis of triglyceride in neuron and neuronal apoptosis via activation of SIRT1‐peroxisome proliferater‐activated receptor (PPAR)γ‐ peroxisome proliferator‐activated receptor coactivator (PGC)‐1α signaling pathway.

**Results:**

The expression levels of SIRT1 or QKI 6, and acetylation level of QKI 6 were decreased in neurons of rat model with CIRI. QKI 6 deacetylated and mediated by SIRT1 that contributed to suppressing the progression of neuronal apoptosis in rat through promoting synthesis of triglyceride in vivo and in vitro via SIRT1‐PPARγ‐PGC‐1α signaling pathway, then inhibiting CIRI.

**Conclusions:**

Our results demonstrated SIRT1 deacetylates QKI 6, the RNA‐binding protein, that affects significantly the synthesis of triglyceride in neurons of CIRI rat model. Moreover, it activated transcription factor peroxisome proliferator‐activated receptorγ coactivator‐1α (PGC‐1α) through post‐transcriptional regulation of the expression of PPARγ, and further enhanced synthesis of triglyceride, thereby restrained the progression of neural apoptosis and CIRI.

## Graphical abstract

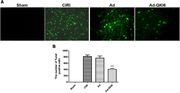

## 1 BACKGROUND

The stroke induced by ischemia of brain remains of high incidence, and it is the considerable reason of eternal death and disability. Therefore, ischemic stroke affects community economy and health (Cuartero et al., 2016). Cerebral ischemia/reperfusion injury (CIRI) is induced by hypoxia and ischemia of brain, and the usual course related to the pathological status of ischemic stroke. Moreover, in the field of ischemia, CIRI is further accentuated by blood flow reperfusion (Jean et al., 1998). The spectrum of complex pathogenesis in CIRI includes inflammation, calcium dysregulation, excitotoxicity, apoptosis, and necrosis. These are a great deal of neurological injuries, which can finally cause the irreversible tissues damage in brain (Moskowitz et al., 2010). CIRI is also the key health problem and challenge, although ischemic stroke is treated with regimens based on the magnificent advances (Catanese et al., 2017; Vuong et al., 2017). Therefore, the refined mechanism of CIRI and the effective therapy strategy of ischemic stroke are required to be identified with more efforts. Many previous investigations explored the effective therapies or medicines to prevent and alleviate ischemic damage (Li et al., 2017; Sharma & Goyal, 2016). Intriguingly, on the basis of different of anti‐inflammation, anti‐apoptosis, and anti‐excitotoxicity mechanisms, CIRI can be relieved in opposition to cerebral ischemia with neuroprotective effects induced by several drugs (H. Wang et al., 2016).

Through regulating the protein modification, sirtuins (SIRT) provide the spectrum of bio‐functions by deacetylating cellular proteins at the post‐translational level (Fujita & Yamashita, 2018). From bacteria to humans, SIRT are widely conserved deacetylased dependent on nicotinamide adenine dinucleotide (NAD) and regulate a series of functions in central neural system (CNS). The obvious evidences demonstrate that SIRT1 maybe a potential strategy for the therapy of neuro‐degenerative diseases. The various neurological disorders and traumatic injury can induce neuronal degeneration usually accompanied with inflammation. Moreover, SIRT1 activation can significantly suppress inflammatory responses and is the sole therapy for controlling both inflammatory cells and neurons (Fujita & Yamashita, 2018).

Quaking (QKI) has different bio‐functions in relation to the stability (Larocque et al., 2005; Pillman et al., 2018; L. Zhao et al., 2006) or translation (de Bruin et al., 2016; Saccomanno et al., 1999) of mRNA, the processing of miRNA (Larocque et al., 2009; Y. Wang et al., 2013), even selective splicing (de Bruin et al., 2016; Fagg et al., 2017; Hall et al., 2013; Hardy, 1998; van der Veer et al., 2013; Zong et al., 2014). We have recently found that suppressed SIRT1 activity increases QKI 6 acetylation. In contrary, the increasing activity of SIRT1 caused deacetylation of QKI 6. Either in neurons knockdown of SIRT1 or neural tissues of SIRT1‐silenced rat, the increased level of QKI 6 acetylation is presented. The previous study indicated that SIRT1 deacetylated QKI, activated FOXO1 and peroxisome proliferater‐activated receptor (PPAR)α, then affected triglyceride (TG) synthesis in NAFLD mouse (Zhang et al., 2019).

Lipid metabolism is associated with a great deal of cellular processes, such as proliferation, differentiation, growth, survival, apoptosis, motility, inflammation, membrane homeostasis, drug resistance, and chemotherapy response. Moreover, bioactive lipid molecules can enhance cell intrinsic apoptotic pathway through activating caspases enzymes and regulating the permeability of mitochondrial membrane. To understand and define the lipid metabolism of neurons is very important to explore the novel strategy for anti‐stroke therapy (Huang & Freter, 2015).

Therefore, the role of QKI 6 mediated by SIRT1 in this work will be elucidated on the pathological process in CIRI model. Ischemic stroke caused by the middle cerebral artery occlusion (MCAO) model subsequently combined with reperfusion (Belayev et al., 1996). The CIRI rat model induced by MCAO was well established and neurons of rats were segregated for identifying the functional characterization of QKI 6 associated with SIRT1 and the activation of PPARγ/peroxisome proliferator‐activated receptor coactivator (PGC)‐1α signaling pathway on the area of neuronal apoptosis in CIRI rat model.

## 2 METHODS

### 2.1 Primary culture for cerebral cortical neurons of rat

Our experiments were performed according to guidelines for use of Laboratory Animals in Fourth Military Medical University (Xi'an, China). Based on previous methods from Oka et al. (2013), cerebral cortical neurons of fetal Sprague–Dawley (SD) rats were isolated from 17‐ to 18‐day‐old embryonic rats.

### 2.2 Ethics approval

All animals were obtained from Department of Laboratory Animal Science, College of Basic Medical Science, Fourth Military Medical University (Xi'an, China). All animal experiments were performed according to the guidelines for the Care and Use of Laboratory Animals established by the Chinese Association for Laboratory Animal Science and approved by the Animal Ethics Committee of Fourth Military Medical University, China. At the end, the mice were over‐anesthetized by increasing the amount of inhaled anesthesia, 0.5 ml/min isoflurane until breathing, and the heartbeat stopped. There was no pain in the entire process. Animal carcasses were handed over to the management department for unified treatment approach.

### 2.3 Oxygen/glucose deprivation

In our work, ischemia was induced with oxygen/glucose deprivation (OGD) (Clarke et al., 1987; Katayama et al., 2017; Karelina et al., 2017). The cytosine arabinoside was used to halt the nonneuronal cell division after 4–5 days in vitro (DIV). Then, at the DIV of 14–15, mature cultures were performed for the experiments. To initiate ischemia, Hypoxic Workstation incubator was used to change the original one.

### 2.4 QKI 6 treatment

The protective role induced by QKI 6 was evaluated. Before exposing to OGD, primary cortical neurons treated with OGD were pretreated with 50, 100, or 200 ng/ml QKI 6 recombinant protein (H00009444‐P01, Abnova), respectively. Meanwhile, the effect of SIRT1 in the protective role caused by QKI 6 was assessed with SRT1720 (a SIRT1 agonist, 50 ng/ml) and Niacinamide (Nic, a selective inhibitor of SIRT1, 10 μM), CIRI was used with DMSO. Then, cell viability was determined for these cultured neurons with regular medium under normoxia after OGD treatment for another 24 h.

### 2.5 Examination injury of neurons

The neuronal injury was quantity evaluated based on LDH activity assay (Karelina et al., 2017). After removing the debris of neurons first, the medium of cultured neurons was centrifuged, and then mixed with reagent of the LDH kit for determining with a spectrophotometer at 340 nm.

### 2.6 Determine death of neurons

The death of neurons was analyzed using TUNEL staining and MTT assay. In MTT assays, neurons were dealt with MTT for 2 h at 37°C, and then released formazan of these neurons was quantified at 560 nm using plate reader. Subsequently, the TUNEL‐stained apoptotic neurons were observed with fluorescence microscope after paraformaldehyde and permeabilized in Triton X‐100.

### 2.7 Analyze the activation of caspase 3

The activation of caspase 3 in neurons was detected with the Activity Assay Kit. Based on manual of the kit, the activation of caspase‐3 was examined using a fluorescence spectrophotometer at *λ*_ex_ 400 nm and *λ*_ex_ 505 nm.

### 2.8 Determination of total TG in primary cerebral cortical neurons of rat

The primary cerebral cortical neurons of rats were cultured in plates after treating with QKI 6 protein. The absorbance at 570 nm of the cell lysate was counted based on standard curve. Then, TG concentrations were normalized with protein concentrations. TGs and cholesterol in the primary cerebral cortical neurons were determined with the infinity TC and TGs kit (TR‐22421, TR13421; Thermo Fisher Scientific, USA), respectively.

### 2.9 Analyze QKI 6 acetylation modification induced by SIRT1

The modified sites of acetylation in QKI6 were predicted with the KA‐predictor, which was an online tool (http://sourceforge.net/p/ka‐predictor).

### 2.10 Adenovirus vector

Ad‐SIRT1, the recombinant adenovirus vector was produced using AdEasy (Ad) Vector System (Stratagene, La Jolla, CA, USA). The infection controls were Ad‐GFP.

Adenovirus‐bacterial recombination system (AdEasy) was used, including pGEM‐3ZF (+) and pAdEasy‐1 pShuttle‐SYN, which was all expression vectors labeled using GFP. The total RNA extracted from cells of rat brain neurons was used for gene amplification of QKI 6, and then it was cloned into vectors based on rat QKI 6 (AF142419.1) sequence in GeneBank. Moreover, the sequence of primer at upward was 5′‐atgcttagtctcagcagcctccgcc‐3′, and the sequence of primer at downward was 5′‐gaccgagccgccaccggcaactaa‐3′. QKI 6 was cloned from primary cultured cortical neurons. The evaluation for concentration of virus and viral vectors titer was performed, and then viral stocks were detected on the replication competent viruses with potential contamination. The concentrations of virus were measured at A260 (Hardy, 1998). The titers of viral vectors Ad‐QKI 6 and Ad‐GFP were 2.5 × 10^11^ and 2.0 × 10^11^ pfu/ml, respectively.

### 2.11 Cell transfection and siRNA of SIRT1

Followed without or with SRT1720 (50 ng/ml), the neurons treated with OGD were followed with treatment of either scrambled or SIRT1 siRNA. The siRNAs of scrambled sequence and SIRT1 siRNA sequence were as follows: scrambled siRNA: 5′‐ACUUUGCUGUAACCCUGUAdTdT‐3′ (sense), 5′‐UACAGGGUUACAGCAAAGUdTdT‐3′ (antisense); SIRT1 siRNA: 5′‐CCUACGCCACCAAUUUCGU‐3′ (sense), 5′‐ACGAAAUUGGUGGCGUAGG‐3′ (antisense).

### 2.12 Immunoprecipitation with acetylated antibody

The total protein of primary cultured cortical neurons induced by OGD was extracted, and then mixed with acetyl lysine antibody and incubated overnight at 4°C. The washed sediments were eluted and mixed with the elution buffer. After centrifugation, the immunoprecipitation (IP) products were assessed using western blot assay with the primary antibody of QKI 6 to determine the content of QKI 6 in the IP products.

### 2.13 Animals and CIRI model caused by MCAO

Our experiment was approved by Institutional Animal Care and Use Committee of Fourth Military Medical University. The SD rats (250–300 g, adult, female) obtained from Beijing Experimental Animal Center (Beijing, China) were used in our experiment. In standard cages, these rats fed with water and food were housed at 22 ± 2°C in a cycle of light/dark (12 h/12 h). Ischemic stroke was induced by MCAO with subsequent reperfusion (Belayev et al., 1996). Rats were anesthetized with urethane (1.4 g/kg, i.p.) (Sigma Chemical Co., St Louis, MO, USA) before surgery. Note that 5.0% urethane in 100% O_2_ until unconscious and anesthesia was maintained with 1.25% urethane in 100% O_2_ at a flow rate of 1 L min^−1^. At first, a nylon filament was inserted in the right carotid artery (diameter, 0.235 mm). After 2 h of advanced occlusion, it was taken out, then blood flow was resupplied with enough reperfusion. The sham rats did not undergo the occlusions but surgery of MCA. After MACO and reperfusion, rat was sacrificed by decollation, and brains tissues and infarct volume were collected.

### 2.14 Adenovirus infection

Four groups were divided averagely from 24 rats: (1) sham, (2) model (CIRI), (3) rat with CIRI and treated with Ad‐QKI 6 (Ad‐QKI 6 group), or (4) empty adenovirus vectors (Ad group). The group of (2) CIRI, (3) Ad‐QKI 6, and (4) Ad were injected with saline, recombinant adenovirus with QKI 6 or saline + adenovirus (empty vector) through the right lateral ventricle for 3 days before ischemia, respectively. The site of injection is based on the brain stereotactic atlas for rats (Clarke et al., 1987). After continuous administration for 8 weeks, rats were MCAO modeled and sacrificed after 24‐h reperfusion. Additionally, brain tissues were subjected to pathological hematoxylin–eosin (HE) and immunohistochemical (IH) stain.

### 2.15 Measurement of infarct volume and neurological deficit

After reperfusion for 24 h, the functions of neurology were assessed with Zea‐Longa score (Clarke et al., 1987; Katayama et al., 2017; Karelina et al., 2017). The volume of infarction was detected using triphenyltetrazolium chloride (TTC) staining. The sacrifice was conducted after reperfusion, and brains were collected and cut into sections (1.0 mm). The sections were fixed with paraformaldehyde after TTC incubation, and then imaged for count of infarct volume using Image Pro Plus (version 16.0).

### 2.16 Western blot

The concentrations of total protein extracted from primary cultured cortical neurons or tissues of brain were quantified using BCA (Bicinchoninic acid assay). Then, protein was electrophoresized with SDS‐PAGE gel (12%), further transferred onto polyvinylidene difluoride membranes. TBS containing nonfat milk was used to block the membranes, then it was incubated with SIRT1 antibodies (1:800; Cell Signaling Technology, Inc., Danvers, MA, USA), QKI 6 (1:2000; Abcam, Cambridge, UK), PGC‐1α (1:1000; Abcam, Cambridge, UK), PPARγ(1:1500; Abcam, Cambridge, UK), or anti‐cleaved caspase‐3 (D175; 1:1200; Cell Signaling Technology, Beverly, MA), and β‐actin mouse mAb (8H10D10; CST #3700; 1:1000; Cell Signaling Technology, Beverly, MA), respectively. Then, those were further incubated using the HRP (horseradish peroxidase)‐conjugated second antibodies (1:1000 dilution; Cell Signaling Technology). In the end, the image of blots was obtained using the electrochemiluminescence detection system.

### 2.17 GFP fluorescence detection

The pentobarbital was used terminally to anesthetize the MCAO model after recovery period; brains were made into 2‐mm thick coronal sections and sliced from the frontal pole after transcardial perfusion with saline. Then, the sections were fixed with 4% formalin and stained with TTC. The area of infarction in each section was analyzed using the software of SigmaScan Pro 5. The signals of GFP were directly obtained using fluorescence microscope of Olympus BX51. The GFP fluorescent cells in each five view fields selected randomly were counted from 100 random cells. The transfection efficiency was calculated and analyzed using GraphPad Prism 5.0 and Image‐ProPlus 6.0.

### 2.18 Analysis of neurological deficit score

The system of score with modification was used to evaluate neurological functional deficit in the MCAO model after recovery period. Neurological deficit score (NDS) of 0%–10% was considered normal, and 100% was considered maximum deficit.

### 2.19 Valuation for volume of infarction area

The tissues of brain in MCAO model were first sectioned, and then the volume of infarction area was valued with TTC method. The captured image was obtained using fluorescence microscope Olympus BX51 (magnification, 200×). In each slice, the infarction area was assessed using image analysis software SigmaScan Pro V5.0 and Photoshop 7.0.

### 2.20 TUNEL staining

At 24 h following MCAO, after fixing with 4% formalin, the brain tissue sections were stained with TUNEL. Five fields of view in the brain tissues sections from contralateral hemisphere were selected randomly. Subsequently, the TUNEL‐stained cells were counted, while the digital photograph was observed from slide of brain tissue. In the end, the obtained NDS and apoptotic index were calculated automatically with GraphPad Prism 5.0.

### 2.21 Statistical analysis

The continuous variables with normally distribute were indicated with mean ± SD (standard deviation). The analysis of statistics was conducted with GraphPad Prism (version 5.0). Student's *t*‐test for comparison of two groups and one‐way analysis of variance (ANOVA) with Kruskal‐Wallis post hoc tests for multiple group comparisons were performed. *p *< .05 was statistically different with significance.

## 3 RESULTS

### 3.1 QKI 6 played a protective role in neuronal death come from OGD

**FIGURE 1:**
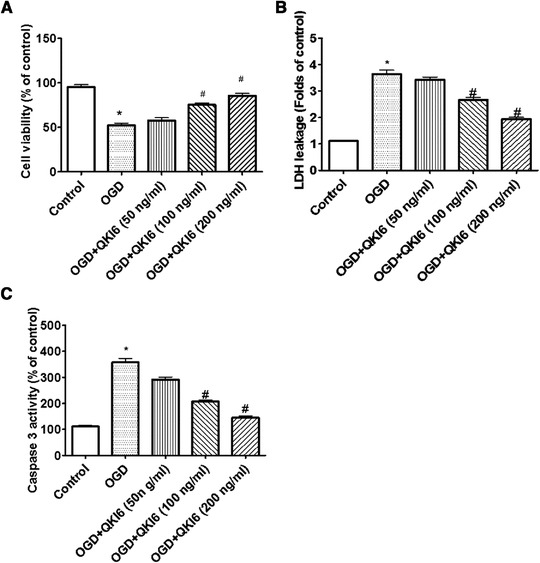
Quaking (QKI) 6 plays a protective role in neuronal death come from oxygen/glucose deprivation (OGD). The results of MTT experiment indicated that the viability of neurons after treatment with OGD obviously decreased, the survived cortical neurons were approximately 55%. However, when neurons were pretreated for 48 h with QKI 6 (50, 100, and 200 ng/ml, respectively) before 24 h exposure to OGD. The viability assay demonstrated that neurons treated with QKI 6 were remarkably better than control neurons and dose dependence (*p *< .01) (A). Further trial results from leakage rate of LDH assay confirmed that it increased obviously after OGD (B). These consequences figured out lactic dehydrogenase (LDH) release could be caused by OGD, which could be also significantly inhibited by QKI 6 and dose dependence (*p *< .001). Moreover, the protective roles of QKI 6 in apoptosis induced by OGD were evaluated with caspase‐3 activity and TUNEL assays. The activity of caspase‐3 obviously increased in cultured cortical neurons treated with OGD (C). But, the QKI 6 treatment could significantly suppress the increased activity of caspase‐3 in a dose‐dependent manner (c). The data are presented as means ± SD from at six independent experiments. *p < .01 (OGD group vs. control group); #*p *< .01 (vs. control group)

At first, the protective effects of QKI 6 on OGD‐induced death of neurons were determined. The results of MTT experiment indicated that the viability of neurons after treatment with OGD obviously decreased, and the survived cortical neurons were approximately 55%. However, when neurons were pretreated for 48 h with QKI 6 protein (50, 100, and 200 ng/ml, respectively) before exposure to OGD (24 h), the viability assay demonstrated that neurons treated with QKI 6 were remarkably better than control neurons with dose dependence (p < .01) (Figure 1A). Further trial results from leakage rate of lactic dehydrogenase (LDH) assay confirmed that it increased obviously after OGD (Figure 1B). These consequences showed LDH release could be caused by OGD, which could be also significantly inhibited by QKI 6 with dose dependence (*p *< .001).

**FIGURE 2:**
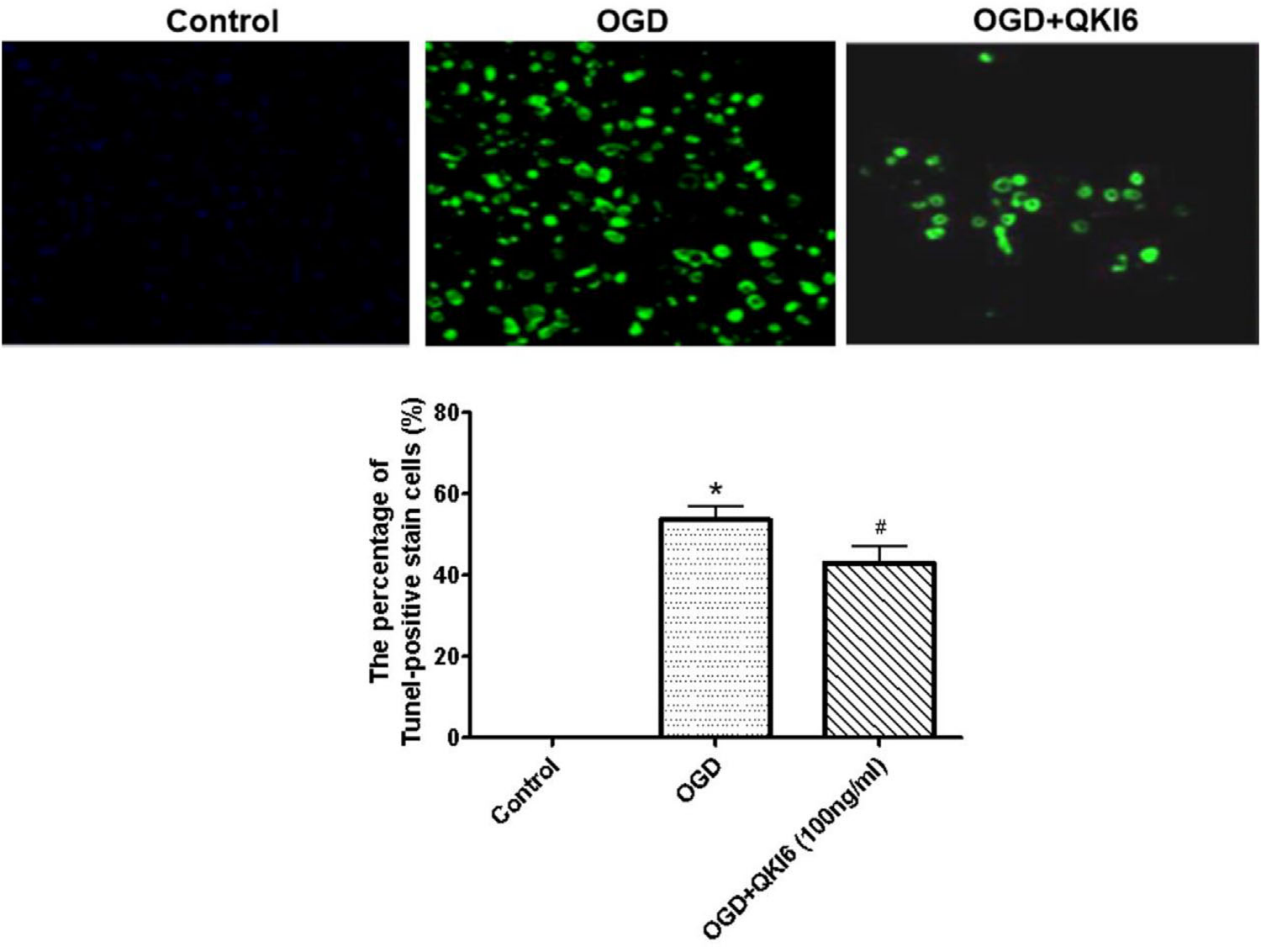
Quaking (QKI) 6 played a protective role in apoptosis of neurons treated with oxygen/glucose deprivation (OGD) with TUNEL assay. The staining of TUNEL increased remarkably in neurons treated with OGD. But, the QKI 6 treatment could significantly reduce the staining of TUNEL in neurons. These results confirmed QKI 6 played a protective role in apoptosis of neurons treated with OGD. The data are presented as mean ± SD from at six independent experiments. *p < .001 (OGD group vs. control group); #*p *< .01 (vs. control group)

Moreover, the protective roles of QKI 6 in apoptosis induced by OGD were evaluated with caspase‐3 activity and terminal‐deoxynucleotidyl transferase mediated nick end labeling (TUNEL) assays. The activity of caspase‐3 obviously increased in cultured cortical neurons treated with OGD (Figure 1C). Meanwhile, the staining of TUNEL increased remarkably in neurons treated with OGD (Figure 2). But, the QKI 6 treatment could significantly suppressed the increased activity of caspase‐3 (Figure 1C) in a dose‐dependent manner and reduced the staining of TUNEL in neurons (Figure 2). These results confirmed QKI 6 played the crucial protective role in apoptosis of neurons treated with OGD.

### 3.2 The lipid metabolic disorders of primary cultured cortical neurons induced by OGD were reversed by QKI 6

**FIGURE 3:**
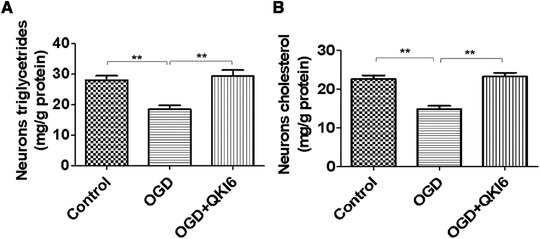
The lipid metabolic disorders of primary cultured cortical neurons induced by oxygen/glucose deprivation (OGD) are reversed by Quaking (QKI) 6. Furthermore, the triglycerides (TGs) (A) and cholesterol (B) content of primary cultured cortical neurons treated with OGD were significantly decreased compared with the controls, but could be restored by QKI 6. It demonstrated that the lipid metabolic disorders of primary cultured cortical neurons induced by OGD could be reversed by QKI 6. The data are presented as means ± SD from at six independent experiments. **p < .01

Furthermore, the TGs (Figure 3A) and cholesterol (Figure 3B) content of primary cultured cortical neurons treated with OGD were significantly decreased compared with the controls, but could be restored by QKI 6. It demonstrated that the lipid metabolic disorders of primary cultured cortical neurons induced by OGD could be reversed by QKI 6 (Figure 3).

### 3.3 SIRT1 regulated the acetylation level of QKI 6

**FIGURE 4:**
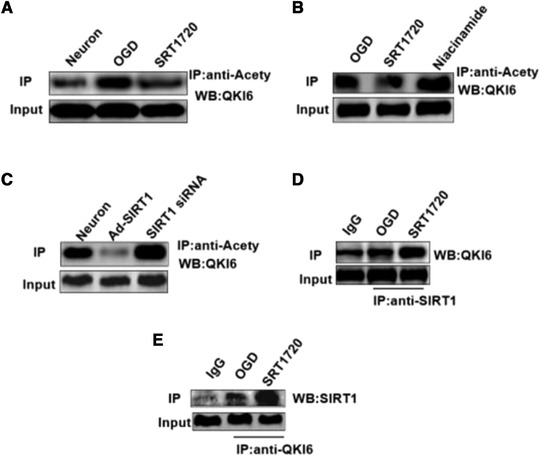
Sirtuins (SIRT)1 regulated the acetylation level of Quaking (QKI) 6. The acetylation level of QKI 6 increased in primary cultured cortical neurons induced by oxygen/glucose deprivation (OGD), but it was reversed by SRT1720 (A). Additionally, the increasing acetylation level of QKI 6 was induced by inhibitor of SIRT1 (Niacinamide), but the QKI 6 acetylation decreased in primary cultured cortical neurons treated with OGD+SRT1720 (B). Furthermore, the increasing acetylation level of QKI 6 was induced by small interfering RNA (siRNA) of SIRT1, but the QKI 6 acetylation decreased in primary cultured cortical neurons treated with Ad‐SIRT1 (C). These results confirmed the protein interaction between SIRT1 and QKI 6, and its level in SRT1720 group was higher than in primary cultured cortical neurons induced by OGD (D and E). (The grouping of gels/blots had been sheared from different gels.)

The acetylation level of QKI 6 increased in primary cultured cortical neurons induced by OGD, but it was reversed by SRT1720 (Figure 4A). Additionally, the increasing acetylation level of QKI 6 was induced by inhibitor of SIRT1 (Niacinamide), but the QKI 6 acetylation decreased in primary cultured cortical neurons treated with OGD+SRT1720 (Figure 4B). Furthermore, the increasing acetylation level of QKI 6 was induced by small interfering RNA (siRNA) of SIRT1, but the QKI 6 acetylation decreased in primary cultured cortical neurons treated with Ad‐SIRT1 (Figure 4C). These results confirmed the protein interaction between SIRT1 and QKI 6, and its level in the SRT1720 group was higher than in primary cultured cortical neurons induced by OGD (Figure 4D and E).

### 3.4 Neuronal SIRT1 regulated the synthesis of TGs in neurons via the QKI 6/PPAR/PGC‐1α signaling pathway in vitro

To evaluate the ability of neuronal SIRT1 to maintain lipid homeostasis, the agonist (SRT1720) and inhibitor (Niacinamide) of SIRT1 were used in this study. Furthermore, adenovirus‐mediated gene repletion of SIRT1 was employed in rat primary neurons, and siRNA of SIRT1 was used to interfere SIRT1 expression.

**FIGURE 5:**
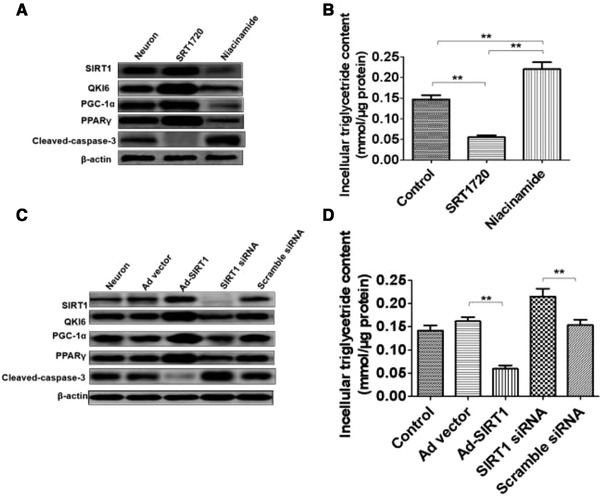
Neuronal sirtuins (SIRT)1 regulated the synthesis of triglycerides (TGs) in neurons via the Quaking (QKI) 6/ peroxisome proliferater‐activated receptor (PPAR)γ/peroxisome proliferator‐activated receptory coactivator (PGC)‐1α signaling pathway in vitro. In primary neurons, SRT1720 upregulated SIRT1 expression, meanwhile expression of QKI 6, PGC‐1α, and PPARγ also increased. However, the SIRT1 expression level was reduced by Niacinamide, which also induced downregulation of QKI 6, PGC‐1α, and PPARγ (A). But, SRT1720 induced downregulation of cleaved‐caspase‐3, and cleaved‐caspase‐3 expression could be upregulated by Niacinamide (A). In addition, the decreased intracellular triglyceride content caused by SRT1720, whereas Niacinamide enhanced the content of TG in primary neurons (B). Additionally, Ad‐SIRT1 enhanced SIRT1, QKI 6, PGC‐1α, and PPARγ expression in primary neurons, which was inhibited by SIRT1 siRNA (C). However, Ad‐SIRT1 induced downregulation of cleaved‐caspase‐3, cleaved‐caspase‐3 expression could be upregulated by siRNA of SIRT1 (C). SRT1720 reduced the content of TGs in primary neurons, and which was promoted by SIRT1 siRNA (D). In addition, SIRT1 mediated the synthesis of TGs in neurons, which was associated with the QKI 6 and the PPARγ/PGC‐1α signaling pathway. The data are presented as means ± SD from at six independent experiments. **p < .01. (The grouping of gels/blots had been sheared from different gels.)

Furthermore, in primary neurons, SRT1720 upregulated SIRT1 expression, meanwhile expression of QKI 6, PGC‐1α, and PPARγ also increased. However, the SIRT1 expression level was reduced by Niacinamide, which also induced downregulation of QKI 6, PGC‐1α, and PPARγ (Figure 5A). But, SRT1720 induced downregulation of cleaved‐caspase‐3, and cleaved‐caspase‐3 expression could be upregulated by Niacinamide (Figure 5A). In addition, the decreased intracellular triglyceride content caused by SRT1720, whereas Niacinamide enhanced the content of TG in primary neurons (Figure 5B). Additionally, Ad‐SIRT1 enhanced SIRT1, QKI 6, PGC‐1α, and PPARγ expression in primary neurons, which was inhibited by SIRT1 siRNA (Figure 5C). But, Ad‐SIRT1 induced downregulation of cleaved‐caspase‐3, and cleaved‐caspase‐3 expression could be upregulated by siRNA of SIRT1 (Figure 5C). SRT1720 reduced the content of TGs in primary neurons, which was promoted by SIRT1 siRNA (Figure 5D). In addition, SIRT1 mediated the synthesis of TGs in neurons, which was associated with the QKI 6 and the PPARγ/PGC‐1α signaling pathway.

### 3.5 Overexpression of QKI 6 protected against transient cerebral ischemia‐induced secondary brain injury

**FIGURE 6:**
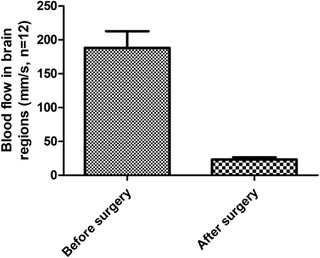
The level of blood flow in the core area. Just after middle cerebral artery occlusion (MCAO), the blood flow of these animal models (*n* = 12) was determined, which decreased to 80%–90% of the baseline level in the core area.

**FIGURE 7:**
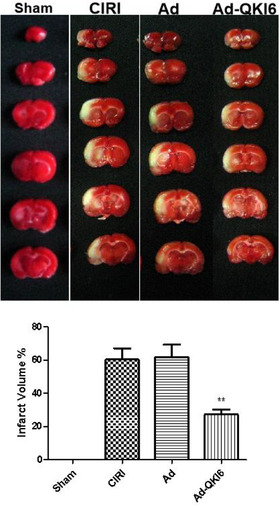
Over‐expression of Quaking (QKI) 6 protected against transient cerebral ischemia‐induced secondary brain injury. Just after middle cerebral artery occlusion (MCAO), the blood flow of these animal models was determined, which decreased to 80%–90% of the baseline level in the core area, it was identified successfully. After MCAO, there was no significant difference in the cerebral blood flow between the two model groups. The representative brain sections showed total volume of infarction and infarction area in each group at 24 h after ischemia. The total volume of infarction in the Ad‐QKI 6 group was 27.31 ± 2.91%, which was significantly smaller than those in the cerebral ischemia/reperfusion injury (CIRI) group (60.36 ± 6.75%) (*p *< .01) and the Ad group (61.80 ± 7.54%) (*p *< .01), respectively. The data are presented as means ± SD from six independent experiments. **p < .01 [Ad‐QKI 6 group (*n* = 6) vs. Ad group (*n* = 6) or CIRI group (*n* = 6)]. (Each group is 6.)

After MCAO, the blood flow of these animal models (*n* = 12) was determined, which decreased to 80%–90% of the baseline level in the core area (Figure 6); it was identified successfully. There was no significant difference in the cerebral blood flow between the two model groups. The representative brain sections showed total volume of infarction and infarction area in each group at 24 h after ischemia (Figure 7). The total volume of infarction in the Ad‐QKI 6 group was 27.31 ± 2.91%, which was significantly smaller than those in the CIRI group (60.36 ± 6.75%) (*p *< .01) and the Ad group (61.80 ± 7.54%) (*p *< .01), respectively.

### 3.6 Overexpression of QKI 6 with recombinant adenovirus reversed postischemic neuronal apoptosis induced by secondary brain damage in transient cerebral ischemia

**FIGURE 8:**
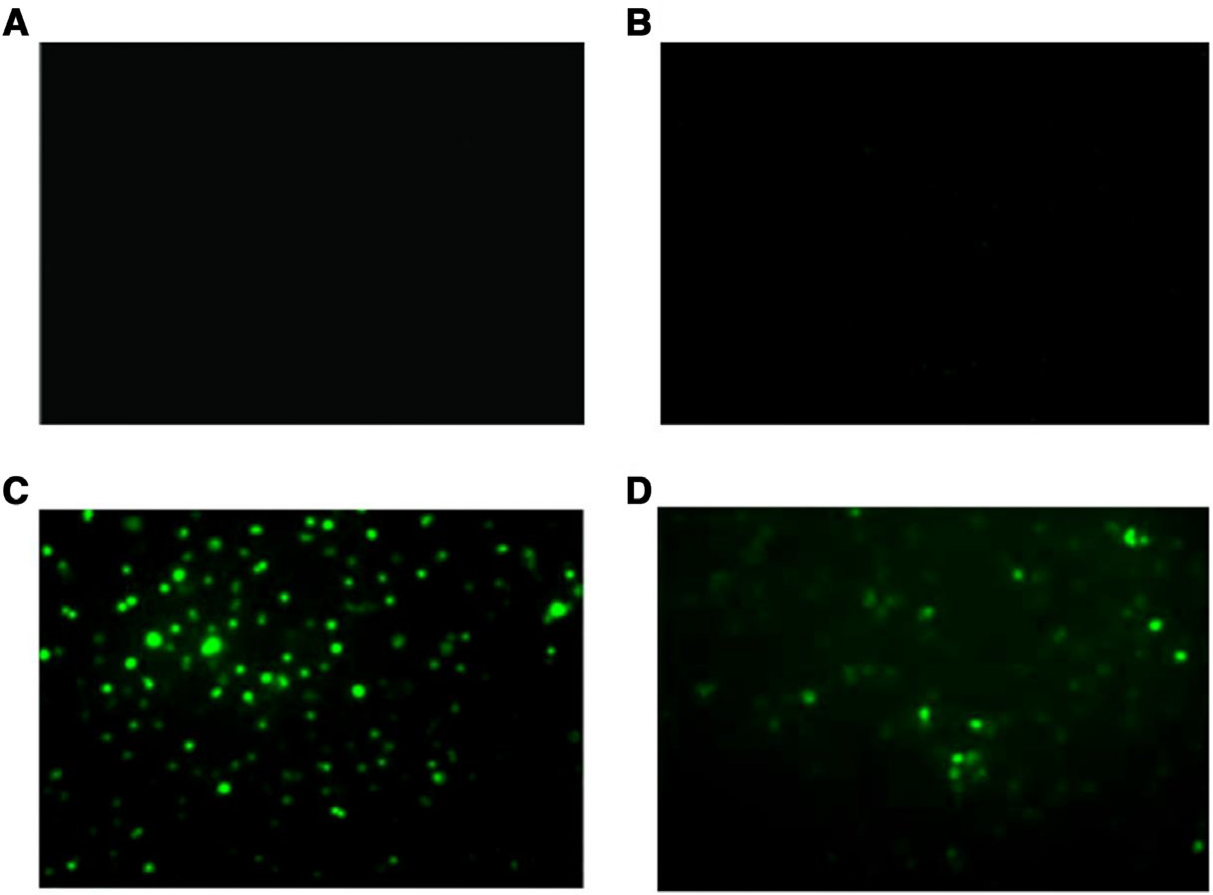
Over‐expression of Quaking (QKI) 6 induced with recombinant adenovirus. In sham (*n* = 6) (A) and cerebral ischemia/reperfusion injury (CIRI) groups (*n *= 6) (B), there was not any green fluorescent protein (GFP) expression. In contrast, expression of GFP was prevalent in the brain tissue (hippocampus, striatum, and cortex penumbra) transfected with Ad (*n* = 6) (C) and Ad‐QKI 6 (*n* = 6) (D), and the transfection rate was about 34.5 ± 3.4%. (Each group is 6.)

**FIGURE 9:**
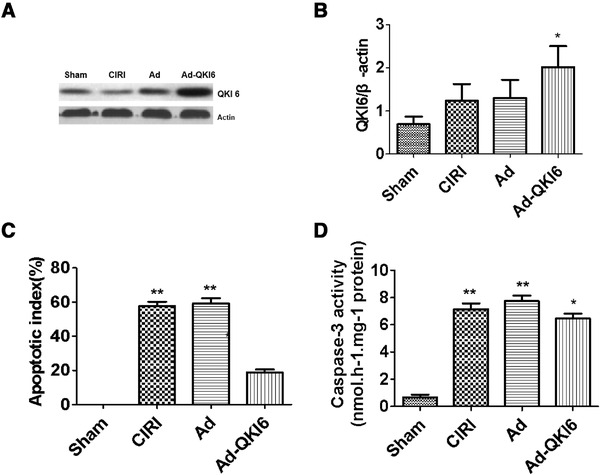
Over‐expression of Quaking (QKI) 6 with recombinant adenovirus reversed post‐ischemic neuronal apoptosis caused by secondary brain damage in transient cerebral ischemia. Then the expression level of QKI 6 was assessed in each group (*n* = 6) with western blot assay. In rats treated with QKI 6, the expression of QKI 6 increased significantly compared with that in cerebral ischemia/reperfusion injury (CIRI) and Ad groups. In the tissues of cerebral cortex, which was obtained from the rats infected with recombinant adenovirus carrying QKI 6 gene, the expression of QKI 6 (A and B) obviously increased. Transient cerebral ischemia injury induced an increased score of apoptotic index (C) at 24 h after middle cerebral artery occlusion (MCAO). Over expression of QKI 6 reversed the apoptosis score compared to the group with transient cerebral ischemia injury. The apoptotic index of the Ad‐QKI 6 group (18.28 ± 0.18%) was remarkably lower than the Ad group (57.68 ± 2.11%) and the CIRI group (58.22 ± 1.96%) (*p *< .001), respectively. The final ordinary pathway of caspase‐dependent apoptosis, caspase‐3 activity of neuron, neuronal apoptosis induced by ischemic was further examined after transfection with QKI 6 gene to identify its specific role. The caspase‐3 activation in brains induced by ischemia/reperfusion reduced remarkably after treatment with QKI 6 gene (D). The data are presented as means ± SD from at six independent experiments. *p < .05 (Ad group or CIRI group vs. sham group); **p < .01 (Ad‐QKI 6 group vs. Ad or CIRI group). (The grouping of gels/blots had been sheared from different gels. Each group is 6.) B: *p < .05; Ad‐QKI 6 group vs. Ad group or CIRI group; it makes sense. C: **p < .01; Ad‐QKI 6 group vs. Ad group or CIRI group; it makes significant sense. D: *p < .05; Ad‐QKI 6 group vs. sham group; it makes sense. **p < .01; Ad‐QKI 6 group vs. Ad group or CIRI group; it makes significant sense

**FIGURE 10:**
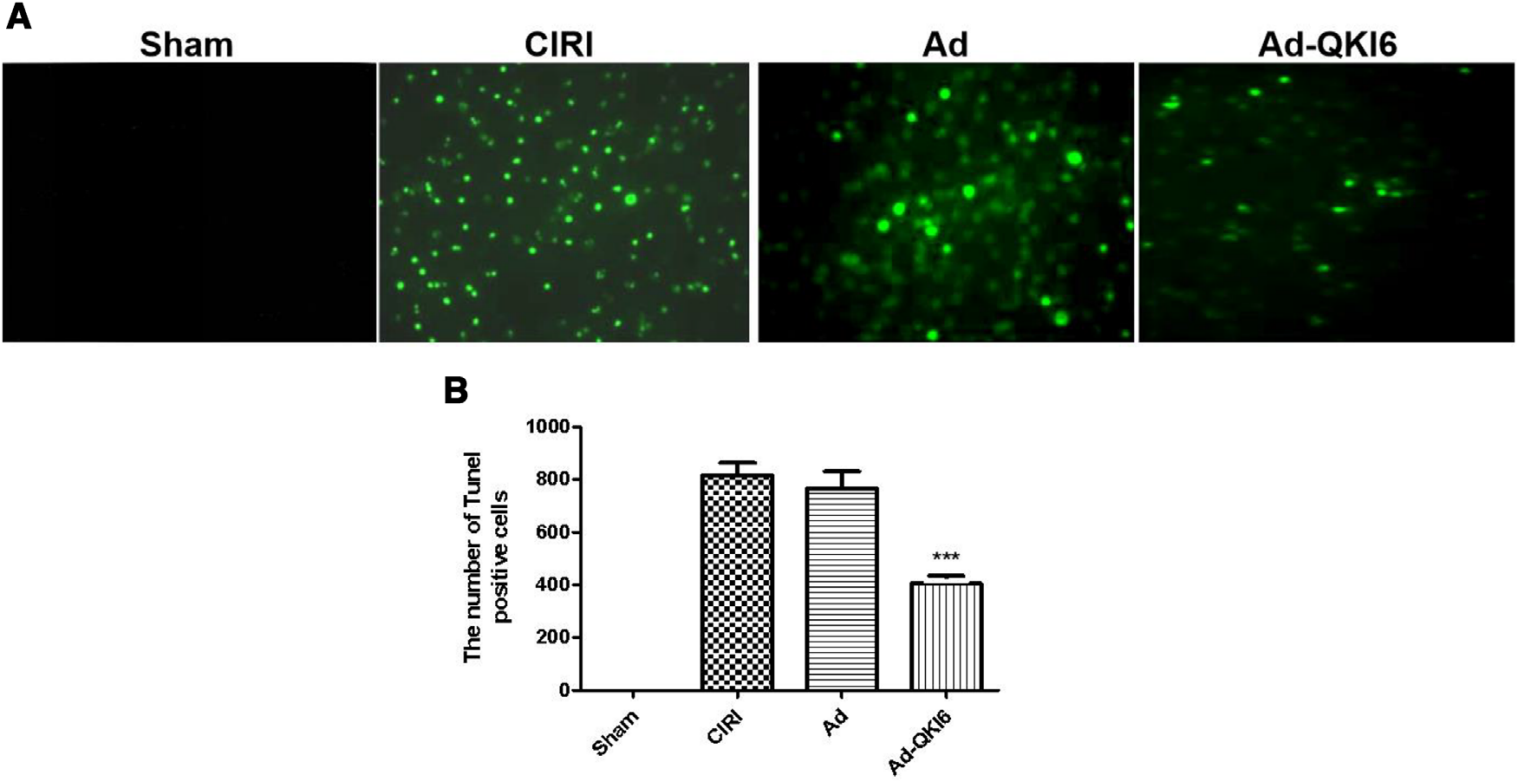
Over‐expression of Quaking (QKI) 6 with recombinant adenovirus reversed post‐ischemic neuronal apoptosis (TUNEL assay) induced by secondary brain damage in transient cerebral ischemia. In brains of the sham group (*n* = 6), the percentage of detected positive cells staining with TUNEL was 0.1%. It demonstrated that the detectable neuronal apoptosis could not be caused by surgical procedure. Conversely, in the tissues from brains of ischemic‐reperfused rats, the nuclei with TUNEL‐positive stain were prevalent. But, it was reduced significantly through treatment with QKI 6 gene. QKI 6 gene over‐expression might play a prohibitive role in post‐ischemic neuronal apoptosis. (Each group is 6.) ***p < .001; Ad‐QKI 6 group vs. Ad group or CIRI group; it makes much more significant sense

In sham (Figure 8A) and CIRI groups (Figure 8B), there was not any green fluorescent protein (GFP) expression. In contrast, expression of GFP was prevalent in the brain tissue (hippocampus, striatum, and cortex penumbra) transfected with Ad (Figure 8C) and Ad‐QKI 6 (Figure 8D), and the transfection rate was about 34.5 ± 3.4%. Then, the expression level of QKI 6 was assessed in each group with western blot assay. In rats treated with QKI 6, the expression of QKI 6 increased significantly compared with CIRI or Ad group. In the tissues of cerebral cortex, which was obtained from the rats infected with recombinant adenovirus carrying QKI 6 gene, the expression of QKI 6 obviously increased (Figure 9A and b). Transient cerebral ischemia injury induced an increased score of apoptotic index at 24 h after MCAO (Figure 9C). Overexpression of QKI 6 reversed the apoptosis score compared to the group with transient cerebral ischemia injury. The apoptotic index of the Ad‐QKI 6 group (18.28 ± 0.18%) was remarkably lower than the Ad group (57.68 ± 2.11%) and the CIRI group (58.22 ± 1.96%) (*p *< .001), respectively. The final ordinary pathway of caspase‐dependent apoptosis, caspase‐3 activity of neuron, neuronal apoptosis induced by ischemic was further examined after transfection with QKI 6 gene to identify its specific role. The caspase‐3 activation in brains induced by ischemia/reperfusion reduced remarkably after treatment with QKI 6 gene (Figure 9D). In brains of the sham group, the percentage of detected positive cells staining with TUNEL was 0.1%. It demonstrated that the detectable neuronal apoptosis could not be caused by surgical procedure. Conversely, in the tissues from brains of ischemic‐reperfused rats, the nuclei with TUNEL‐positive stain were prevalent. But, it was reduced significantly through treatment with QKI 6 gene (Figure 10). QKI 6 gene overexpression might play a prohibitive role in postischemic neuronal apoptosis.

### 3.7 Overexpression of QKI 6 with recombinant adenovirus reverses behavioral impairments induced by secondary brain damage in transient cerebral ischemia via PPARγ/PGC‐1α signaling pathway

**FIGURE 11:**
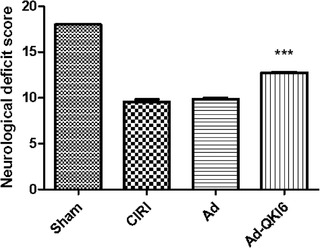
Over‐expression of Quaking (QKI) 6 with recombinant adenovirus reversed behavioral impairments induced by secondary brain damage in transient cerebral ischemia. Transient cerebral ischemia damage could reduce the neurological score at 24 h after middle cerebral artery occlusion (MCAO). Over‐expression of QKI 6 may reverse the score compared to the transient cerebral ischemia injury group. The neurological score of the Ad‐QKI 6 group (12.45 ± 0.18%) were higher than that in the Ad group (9.96 ± 0.09%) and the CIRI group (9.82 ± 0.17%) (*p *< .001), respectively. Over‐expression of QKI 6 gene played a potential protective role against secondary brain damage of cerebral cortex induced by transient cerebral ischemia after MCAO, meanwhile may restore neurological/behavioral impairment. The data are presented as means ± SD from at six independent experiments. ***p < .001 [Ad‐QKI 6 group (*n* = 6) vs. Ad group (*n* = 6) or cerebral ischemia/reperfusion injury (CIRI) group (*n* = 6)]. (Each group is 6.)

Transient cerebral ischemia damage could reduce the neurological score (Figure 11) at 24 h after MCAO. Overexpression of QKI 6 may reverse the score compared to transient cerebral ischemia injury group. The neurological score of the Ad‐QKI 6 group (12.45 ± 0.18%) was higher than that in the Ad group (9.96 ± 0.09%) and the CIRI group (9.82 ± 0.17%) (*p *< .001), respectively. Overexpression of QKI 6 gene played a potential protective role against secondary brain damage of cerebral cortex induced by transient cerebral ischemia after MCAO; additionally, it may restore neurological/behavioral impairment.

**FIGURE 12:**
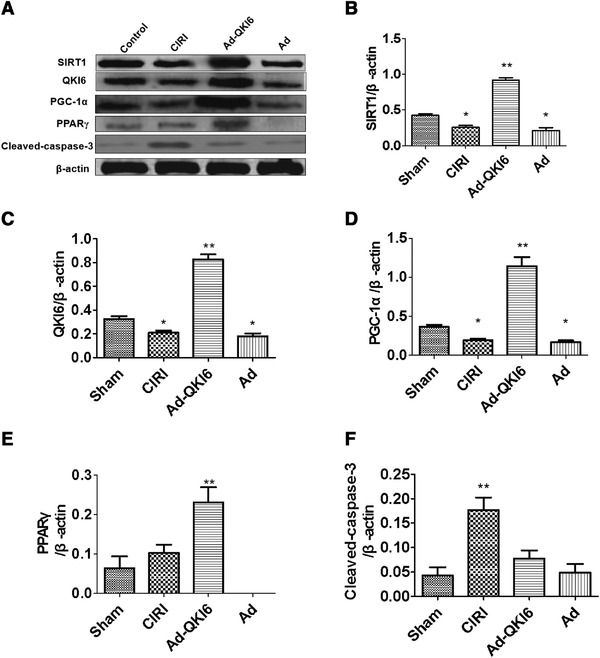
Overexpression of Quaking (QKI) 6 with recombinant adenovirus reversed cerebral ischemia/reperfusion injury (CIRI) induced by secondary brain damage in transient cerebral ischemia via peroxisome proliferater‐activated receptor (PPAR)γ/peroxisome proliferator‐activated receptorγ coactivator (PGC)‐1α signaling pathway. The results of western blot assay demonstrated that the expression level of sirtuins (SIRT)1, QKI 6, PPARγ, and PGC‐1α decreased in model rat, but it was reversed by QKI 6. It demonstrated that neuronal SIRT1 regulated expression of QKI 6 via SIRT1‐PPARγ‐PGC‐1α signaling pathway axis. However, over‐expression of OKI 6 with its recombinant adenovirus induced downregulation of cleaved‐caspase‐3 in CIRI model comes from secondary brain damage of cerebral ischemia. (The grouping of gels/blots had been sheared from different gels. Each group is 6.) B: *p < .05; sham group versus Ad group or CIRI group; it makes sense. **p < .01; Ad‐QKI 6 group versus Ad group or CIRI group; it makes significant sense. C: *p < .05; sham group versus. Ad group or CIRI group; it makes sense. **p < .01; Ad‐QKI 6 group versus Ad group or CIRI group; it makes significant sense. D: *p < .05; sham group versus Ad group or CIRI group; it makes sense. **p < .01; Ad‐QKI 6 group versus Ad group or CIRI group; it makes significant sense. E; **p < .01; Ad‐QKI 6 group versus Ad group or CIRI group; it makes significant sense. F: **p < .01; Ad‐QKI 6 group versus Ad group or CIRI group; it makes significant sense

The results of western blot assay demonstrated that the expression level of SIRT1, QKI 6, PPARγ, and PGC‐1α decreased in model rat, but it was reversed by QKI 6 (Figure 12). However, overexpression of QKI 6 with its recombinant adenovirus‐induced downregulation of cleaved‐caspase‐3 in CIRI model comes from secondary brain damage of transient cerebral ischemia. It demonstrated that neuronal SIRT1 regulated expression of QKI 6 via SIRT1‐PPARγ‐PGC‐1α signaling pathway axis.

## 4 DISSCUSION

RNA‐binding protein‐mediated posttranscriptional regulation is rarely studied in neuronal metabolism. STAR family member QKI is an RNA‐binding protein that produces multiple isoforms in human body and shows various expression patterns in several kinds of tissue cells (Larocque et al., 2005). Among them, QKI 6 is mainly located in the nucleus but can also shuttle to the cytoplasm (Saccomanno et al., 1999; L. Zhao et al., 2006), while QKI 6 and QKI 7 are mainly distributed in the cytoplasm. By binding to the specific recognition element (SRE) of the 3′UTR of mRNA (de Bruin et al., 2016; Y. Wang et al., 2013), QKI plays a key role in regulating the cytoplasmic/nuclear localization, stability, and translation efficiency of mRNA (Larocque & Richard, 2005; Hall et al., 2013). We found that QKI is expressed in primary neurons extracted from cerebral cortex of rat, and the mainly expressed isomer was QKI 6, but its functional studies in the brain had not been reported.

SIRT1 is mediated by posttranslational regulation, and many transcription factors such as FOXO1, SREBP, ChREBP, PPARα, PPARγ, and PGC1α are mediated through SIRT1 at the transcriptional level (Sonnemann et al., 2016). QKI is an RNA‐binding protein whose mediated regulation is posttranscriptional regulation of RNA levels. Specific to RNA sequence binding, QKI is widely involved in variable splicing, subcellular localization, stability maintenance, and translational regulation of RNA.

The major reasons of death and long‐term disability worldwide include stroke. It is related to obvious socioeconomic and clinical implications, especially the need of effective therapies. In practice, stroke is a pathological process in which multifactors are involved, mainly related to oxidative stress, inflammation, calcium overload, brain edema, cell apoptosis, and other relevant events (Voelker, 2016). Transient ischemic attacks (TIAs) are known as brief ischemic episodes according to clinical practice and have been studied for over two decades (Murry et al., 1986; Phan et al., 2016). The tolerance could be induced by TIAs through elevating the threshold of human brain tissue vulnerability (Wegener et al., 2004). It is the critical response for neuroprotection and the potential molecular mechanisms. Till now, there are no clinically effective therapies for stroke, in spite of a great number of studies based on experimental neuroprotective compounds and animal model demonstrated the promising results. Investigating potential molecular mechanisms associated with TIAs may discover the novel targets on the prevention and treatment for stroke (Wilson et al., 2016; H. D. Zhao et al., 2013).

Therefore, rat neurons were first isolated in our study and used to characterize the roles of function and molecular mechanisms of QKI 6, whether QKI 6 protects against neuronal death induced by OGD. OGD induced remarkably LDH release of neurons, but neurons treated with QKI 6 followed with OGD were remarkably better than control neurons only treated with OGD. The various doses of QKI 6 suppressed obviously LDH release with dose dependence. Moreover, QKI 6 provided significantly protective effects on neuronal apoptosis induced by OGD, which was identified by the results of caspase‐3 activity and TUNEL assays. Meanwhile, the lipid metabolic disorders of primary cultured cortical neurons induced by OGD could be reversed by QKI 6.

Further investigation had been conducted to confirm the molecular mechanism of QKI 6. The online local tool of KA predictor (http://sourceforge.net/p/ka‐predictor) was used to predict the acetylation modification site of QKI 6. Therefore, SIRT1 was predicted that it may acetylate QKI 6. We found that the acetylation level of QKI 6 increased in primary cultured cortical neurons induced by OGD, but it was reversed by SRT1720 (the activator of SIRT1). Additionally, the acetylation level of QKI 6 increased in primary cultured cortical neurons induced by OGD, but it was reversed by SRT1720. Additionally, the increasing acetylation level of QKI 6 was induced by inhibitor of SIRT1 (Niacinamide), but the QKI 6 acetylation decreased in primary cultured cortical neurons treated with OGD+SRT1720. Furthermore, the increasing acetylation level of QKI 6 was induced by siRNA of SIRT1, but the QKI 6 acetylation decreased in primary cultured cortical neurons treated with Ad‐SIRT1. These results confirmed the protein interaction between SIRT1 and QKI 6, and its level in the SRT1720 group was higher than in primary cultured cortical neurons induced by OGD.

To evaluate the ability of neuronal SIRT1 to maintain lipid homeostasis, the agonist (SRT1720) and inhibitor (Niacinamide) of SIRT1 were used in this study. Furthermore, adenovirus‐mediated gene repletion of SIRT1 was employed in rat primary neurons, and siRNA of SIRT1 was used to suppress SIRT1 expression. These experiments identified that in primary neurons, SIRT1 mediates the synthesis of TGs in the OGD model, which is associated with the QKI 6 and the PPARγ/PGC‐1α signaling pathway.

Besides, the MCAO‐induced CIRI rat model was well established and used to characterize the effects of QKI 6 mediated by SIRT1 on synthesis of triglyceride in neuron and neuronal apoptosis via activation of SIRT1‐PPARγ‐PGC‐1α signaling pathway. It demonstrated that overexpression of QKI 6 with recombinant adenovirus protected against transient cerebral ischemia‐induced by secondary brain injury, and reversed postischemic neuronal apoptosis and behavioral impairments induced by secondary brain damage in transient cerebral ischemia via PPARγ/PGC‐1 α signaling pathway.

## 5 CONCLUSIONS

Our results demonstrated SIRT1 deacetylates QKI 6, the RNA‐binding protein, that affects significantly the synthesis of triglyceride in neurons of CIRI rat model. Moreover, it activated transcription factor PGC‐1α through posttranscriptional regulation of the expression of PPARγ, and further enhanced synthesis of triglyceride, thereby restrained the progression of neural apoptosis and CIRI.

## CONFLICT OF INTEREST

The authors declare that they have no conflict of interest.

## FUNDING INFORMATION

National Natural Science Foundation of China; Grant Number: 81771469; Shanxi Province Social Development Science and Technology Attack Project; Grant Number: 2016SF‐132; Logistics Research Program of Chinese People's Liberation Army; Grant Number: CJN14J005; Science and Technology Project of Louyang, Henan Province, China; Grant Number: 1603002A‐13.

## AUTHOR CONTRIBUTIONS

Rui Liu and Hongzeng Li contributed to the conception of the study. Rui Liu, Jingyuan Deng and Qunqiang Wu contributed significantly to analysis and manuscript preparation; Chunhua Liao, Qun Xiao, and Qi Chang performed the data analyses and wrote the manuscript; Rui Liu and Qi Chang helped perform the analysis with constructive discussions.

## ETHICS APPROVAL AND CONSENT TO PARTICIPATE

Our experiments were performed according to Guidelines for use of Laboratory Animals in Fourth Military Medical University (Xi'an, China).

### PEER REVIEW

The peer review history for this article is available at https://publons.com/publon/10.1002/brb3.2271.

## Data Availability

All data generated or analyzed during this study are included in this published article.
